# Influenza and COVID-19 co-infection and vaccine effectiveness against severe cases: a mathematical modeling study

**DOI:** 10.3389/fcimb.2024.1347710

**Published:** 2024-03-04

**Authors:** Jingyi Liang, Yangqianxi Wang, Zhijie Lin, Wei He, Jiaxi Sun, Qianyin Li, Mingyi Zhang, Zichen Chang, Yinqiu Guo, Wenting Zeng, Tie Liu, Zhiqi Zeng, Zifeng Yang, Chitin Hon

**Affiliations:** ^1^ Department of Engineering Science, Faculty of Innovation Engineering, Macau University of Science and Technology, Taipa, Macao SAR, China; ^2^ Respiratory Disease AI Laboratory on Epidemic and Medical Big Data Instrument Applications, Faculty of Innovation Engineering, Macau University of Science and Technology, Macao, Macao SAR, China; ^3^ Guangzhou Key Laboratory for Clinical Rapid Diagnosis and Early Warning of Infectious Diseases, KingMed School of Laboratory Medicine, Guangzhou Medical University, Guangzhou, China; ^4^ State Key Laboratory of Respiratory Disease, National Clinical Research Center for Respiratory Disease, Guangzhou Institute of Respiratory Health, The First Affiliated Hospital of Guangzhou Medical University, Guangzhou, Guangdong, China; ^5^ Guangzhou Laboratory, Guangzhou, Guangdong, China

**Keywords:** SARS-CoV-2, influenza, co-infection, vaccination, compartmental model

## Abstract

**Background:**

Influenza A virus have a distinctive ability to exacerbate SARS-CoV-2 infection proven by in vitro studies. Furthermore, clinical evidence suggests that co-infection with COVID-19 and influenza not only increases mortality but also prolongs the hospitalization of patients. COVID-19 is in a small-scale recurrent epidemic, increasing the likelihood of co-epidemic with seasonal influenza. The impact of co-infection with influenza virus and SARS-CoV-2 on the population remains unstudied.

**Method:**

Here, we developed an age-specific compartmental model to simulate the co-circulation of COVID-19 and influenza and estimate the number of co-infected patients under different scenarios of prevalent virus type and vaccine coverage. To decrease the risk of the population developing severity, we investigated the minimum coverage required for the COVID-19 vaccine in conjunction with the influenza vaccine, particularly during co-epidemic seasons.

**Result:**

Compared to the single epidemic, the transmission of the SARS-CoV-2 exhibits a lower trend and a delayed peak when co-epidemic with influenza. Number of co-infection cases is higher when SARS-CoV-2 co-epidemic with Influenza A virus than that with Influenza B virus. The number of co-infected cases increases as SARS-CoV-2 becomes more transmissible. As the proportion of individuals vaccinated with the COVID-19 vaccine and influenza vaccines increases, the peak number of co-infected severe illnesses and the number of severe illness cases decreases and the peak time is delayed, especially for those >60 years old.

**Conclusion:**

To minimize the number of severe illnesses arising from co-infection of influenza and COVID-19, in conjunction vaccinations in the population are important, especially priority for the elderly.

## 1 Introduction

With the relaxation of COVID-19 policies worldwide, the management of COVID-19 has now entered a phase of normalization. The previously implemented Public Health and Social Measures (PHSM) during the COVID-19 pandemic also had a preventive effect on the influenza epidemic, which may result in an immune gap among the population (Ali et al., 2022; Yang et al., 2022). The immune evasion ability of SARS-CoV-2 is well recognized by encoding seven accessors (Zandi et al., 2022), and ORF9c and ORF10 play key roles (Zandi, 2022). Besides, highly variable characteristics of the influenza virus led to continuous antigen drift and change of susceptible populations, causing repeated global influenza epidemics Therefore, the co-epidemics of COVID-19 and influenza in the coming years are inevitable.

Since the early stages of the pandemic, several studies have emerged mixed infections involving SARS-CoV-2 and influenza (Yang et al., 2022; Rezaee et al., 2023). A meta-analysis indicated that the prevalence of influenza co-infection among COVID-19 patients is 0.8%, with a frequency of 4.5% in Asia and 0.4% in the Americas (Kim et al., 2020). In the initial outbreak of COVID-19 in Wuhan, co-infections of SARS-CoV-2 and influenza viruses were highly common, with a shift from IBV to IAV in terms of the co-infection viral types (Yue et al., 2020).

Currently, there is a large amount of clinical evidence indicating that co-infection with COVID-19 and influenza increases the risk of patient mortality and prolongs hospitalization (Dadashi et al., 2021; Garg et al., 2022; Cong et al., 2022; Fahim et al., 2022; Musuuza et al., 2021). In patients with co-infections, the risk of death is around twice as high as in those with COVID-19 infection alone (Stowe et al., 2021). These findings are also supported by basic experiments, as laboratory studies have confirmed the synergistic effect of influenza on the infection of SARS-CoV-2. Research by Bai et al. suggests that pre-infection with influenza A virus (IAV) significantly enhances the infection of SARS-CoV-2 in various cell types (Swets et al., 2022). Furthermore, co-infection with SARS-CoV-2 and IAV noticeably reduces the levels of total IgG and neutralizing antibodies against both IAV and SARS-CoV-2 (Kim et al., 2022).

Recent studies have shown that there is a certain degree of cross-protection between COVID-19 and influenza, and vaccines are effective in preventing co-infections (Alosaimi et al., 2021; Varshney et al., 2023). Influenza vaccination has been proven to reduce the risk of SARS-CoV-2 infection (Marín-Hernández et al., 2021; Wang et al., 2021). For example, Wilcox et al. found a significant correlation between receiving influenza vaccination and a decrease in hospitalization or all-cause mortality, with a 24% reduction in all-cause mortality (adjusted odds ratio: 0.76, 95% confidence interval: 0.64-0.90) (Wilcox et al., 2021). However, studies have revealed that older adults and high-risk individuals with chronic diseases have a lower willingness to receive influenza vaccination (Katsiroumpa et al., 2023). Early research has shown a link between influenza vaccination among older adults and a decrease in COVID-19 hospitalization rates and mortality (Candelli et al., 2021; Yang et al., 2021). Therefore, promoting vaccine uptake among specific populations is warranted.

Regarding the issue mentioned above, the World Health Organization (https://www.who.int/tools/flunet) has started publishing data on the positivity rates of influenza and COVID-19. Multiple countries, including the United States, Israel, and India, have advised paying special attention to the issue of co-infection. So far, the world has experienced three waves of COVID-19, and the Omicron variant is predominant in the fourth wave (Gao et al., 2022). SARS-CoV-2 is constantly evolving (Bedford et al., 2015), and the Influenza virus is experiencing interchangeable strain circulation. The impact of such diverse co-pandemics on population health is still unknown.、

Current models of co-infection between COVID-19 and influenza (Du et al., 2022; Pinky and Dobrovolny, 2022; Ojo et al., 2022) have not considered the combination of types or the influence of vaccination and cross-immunity. Therefore, optimizing models to address these research gaps would be crucial for accurately predicting the severity of co-infection and for the development of effective prevention and control strategies.

## 2 Materials and methods

To quantify the mutual influence of the two viruses, we developed a model to illustrate how the interactions of the viruses at the individual level affect the epidemiological situation at the population level. This modeling study relies on publicly available aggregated data only. As such, institutional review and informed consent are waived by the Institutional Review Board of the first affiliated hospital of Guangzhou Medical University (Guangzhou, China)

### 2.1 Model construction

**Figure 1:**
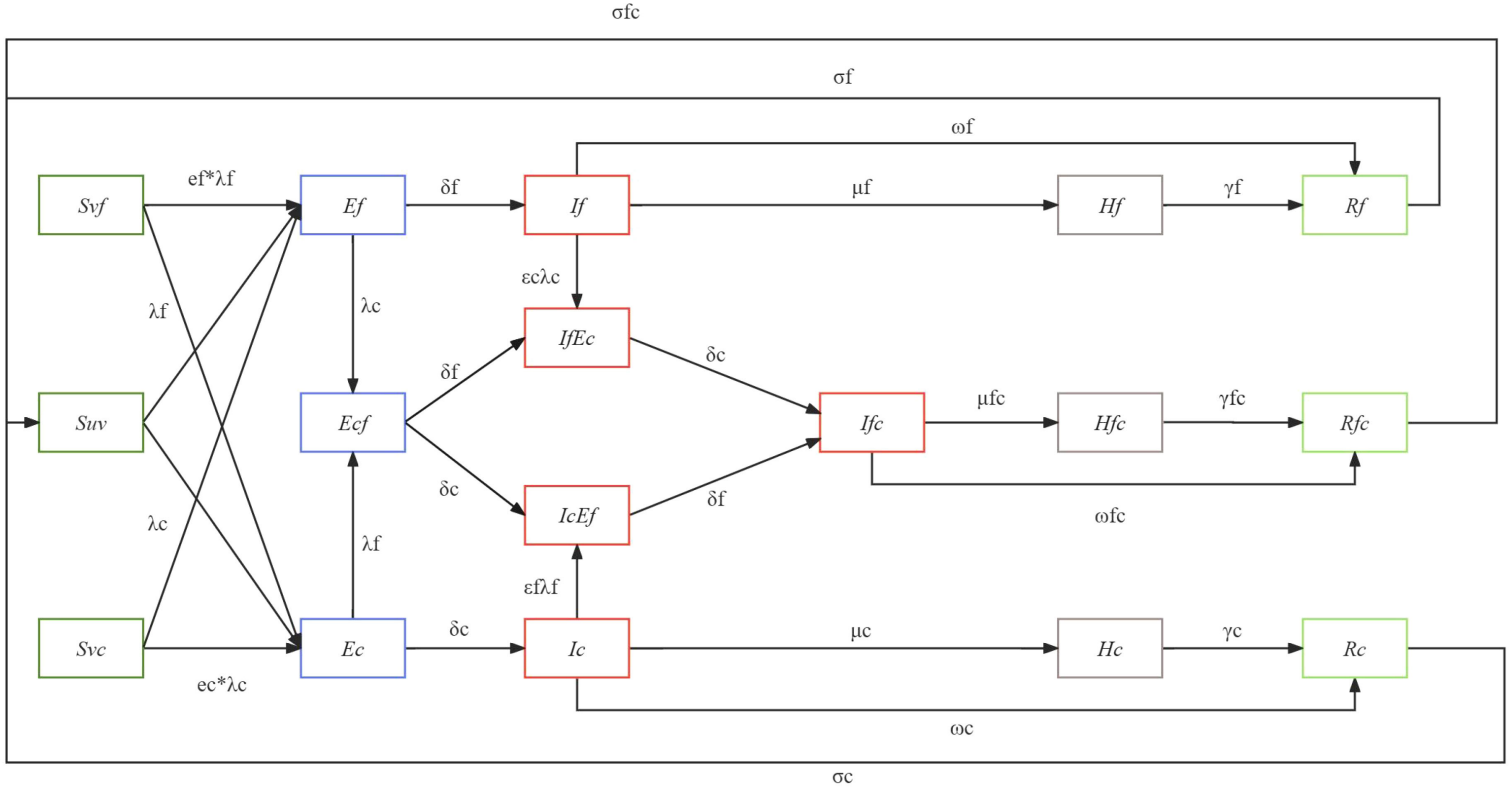
The model diagram of the developed coinfection model.

Based on epidemiological data, mathematical models can be used to estimate the impact of interactions on transmission and help develop prevention and control strategies (Chang et al., 2021). Considering that statistical method could not formalize the transmission process or biological mechanism, and the interaction mechanism cannot be determined nor the strength of interaction quantified. However, compartment model could dissect the cause and effect of the different components (Opatowski et al., 2018). Therefore, we developed an age-specific compartment model to illustrate the transition patterns of different population groups during a simultaneous outbreak of influenza and COVID-19. The model diagram is given in **Figure 1**.

In this model, the population is categorized into susceptible (S), exposed (E), infectious (I), recovered (R), and severe cases (H). Within the susceptible group (S), we consider the vaccine effectiveness (VE) against infection, subsequently dividing them into those protected by the influenza vaccine (Svf), those protected by the COVID-19 vaccine (Svc), and those unprotected by any vaccine (Suv). The probability of infection varies among these susceptible groups, influenced by two vaccine protection parameters (ef, ec), representing the protection rate of the influenza vaccine against influenza infection (ef) and the protection rate of the COVID-19 vaccine against COVID-19 infection (ec). These susceptible individuals may contract either influenza, COVID-19, or both, transitioning to the exposed group (E) at infection rates (λf, λc). Considering that individuals can be solely infected or co-infected, the exposed population (E) is subdivided into those exposed to influenza (Ef), those exposed to COVID-19 (Ec), and those co-exposed to both (Ecf). For those exposed solely to either influenza or COVID-19 (Ef, Ec), they transition to the infectious group (If, Ic) after an incubation period (δf, δc). As for the co-exposed group (Ecf), they evolve into two infectious states: those infectious for influenza but exposed to COVID-19 (IfEc) and those infectious for COVID-19 but exposed to influenza (IcEf), subsequently becoming co-infected with both (Ifc). Considering the pre-infection may enhance the susceptibility of the other virus, we have introduced two key parameters, represent the change in susceptibility to SARS-CoV-2 in patients with influenza virus infection, and represent the change in susceptibility to influenza virus in patients with SARS-CoV-2 infection. Infectious individuals (If, Ic, Ifc) will transit to the recovered group (Rf, Rc, Rfc) over a recovery period (ωf, ωc, ωfc). However, given the possibility of disease exacerbation, some will progress to severe cases (Hf, Hc, Hfc) with transition probabilities μf, μc, μfc, representing severity rates for influenza, COVID-19, and co-infections respectively. We also account for vaccine-mediated protection against severity, incorporating three parameters: the protection rate of the vaccine against severe influenza (βf), against severe COVID-19 (βc), and severe co-infection (βfc). As our model does not consider mortality, these severe cases will transition to the recovered group (Rf, Rc, Rfc) over the recovery period (γf, γc, γfc). Lastly, given the realistic scenario where immunity wanes leading to potential re-infections, those recovered will gradually lose their immunity and revert to the susceptible state (S) with waning immunity probabilities of σf, σc, σfc.

The model equations are as follows:


$$\left\{ \begin{array}{l} \frac{dS_{uv}(t)}{dt}=-\frac{\left(\lambda_{f}*S_{uv}(t)*(I_{f}(t)+I_{fEc}(t)+I_{fc}V(t))+\lambda_{c}*S_{uv}(t)*(I_{c}(t)+I_{cEf}(t)+I_{fc}V(t))\right)}{N}+\sigma_{f}*R_{f}(t)+\sigma_{c}*R_{c}(t)+\sigma_{fc}*R_{fc}(t)\\ \frac{dS_{vf}(t)}{dt}=-\frac{\left(\lambda_{f}*e_{f}*S_{vf}(t)*(I_{f}(t)+I_{fEc}(t)+I_{fc}V(t))+\lambda_{c}*S_{vf}(t)*(I_{c}(t)+I_{cEf}(t)+I_{fc}V(t))\right)}{N}\\ \frac{dS_{vc}(t)}{dt}=-\frac{\left(\lambda_{f}*S_{vc}(t)*(I_{f}(t)+I_{fEc}(t)+I_{fc}V(t))+\lambda_{c}*e_{c}*S_{vc}(t)*(I_{c}(t)+I_{cEf}(t)+I_{fc}V(t))\right)}{N}\\ \frac{dE_{f}(t)}{dt}=\frac{\lambda_{f}*S_{uv}(t)*(I_{f}(t)+I_{fEc}(t)+I_{fc}V(t))+\lambda_{f}*e_{f}*S_{vf}(t)*(I_{f}(t)+I_{fEc}(t)+I_{fc}V(t))+\lambda_{f}*S_{vc}(t)*(I_{f}(t)+I_{fEc}(t)+I_{fc}V(t))-\lambda_{c}*E_{f}(t)*(I_{c}(t)+I_{cEf}(t)+I_{fc}V(t))}{N}-\delta_{f}*E_{f}(t)\\ \frac{dE_{c}(t)}{dt}=\frac{\lambda_{c}*S_{uv}(t)*(I_{c}(t)+I_{cEf}(t)+I_{fc}V(t))+\lambda_{c}*S_{vf}(t)*(I_{c}(t)+I_{cEf}(t)+I_{fc}V(t))+\lambda_{c}*e_{c}*S_{vc}(t)*(I_{c}(t)+I_{cEf}(t)+I_{fc}V(t))-\lambda_{f}*E_{c}(t)*(I_{f}(t)+I_{fEc}(t)+I_{fc}V(t))}{N}-\delta_{c}*E_{c}(t)\\ \frac{dE_{fc}(t)}{dt}=\frac{\lambda_{f}*E_{c}(t)*(I_{f}(t)+I_{fEc}(t)+I_{fc}V(t))+\lambda_{c}*E_{f}(t)*(I_{c}(t)+I_{cEf}(t)+I_{fc}V(t))}{N}-(\delta_{c}+\delta_{f})*E_{fc}(t)\\ \frac{dI_{f}(t)}{dt}=\delta_{f}*E_{f}(t)-\frac{\varepsilon_{c}*\lambda_{c}*I_{f}(t)*(I_{c}(t)+I_{cEf}(t)+I_{fc}V(t))}{N}-\mu_{f}*I_{f}(t)-\omega_{f}*I_{f}(t)\\ \frac{dI_{c}(t)}{dt}=\delta_{c}*E_{c}(t)-\frac{\varepsilon_{f}*\lambda_{f}*I_{c}(t)*(I_{f}(t)+I_{fEc}(t)+I_{fc}V(t))}{N}-\mu_{c}*I_{c}(t)-\omega_{c}*I_{c}(t)\\ \frac{dI_{fEc}(t)}{dt}=\delta_{f}*E_{fc}(t)+\frac{\varepsilon_{c}*\lambda_{c}*I_{f}(t)*(I_{c}(t)+I_{cEf}(t)+I_{fc}V(t))}{N}-\delta_{c}*I_{fEc}(t)\\ \frac{dI_{cEf}(t)}{dt}=\delta_{c}*E_{fc}(t)+\frac{\varepsilon_{f}*\lambda_{f}*I_{c}(t)*(I_{f}(t)+I_{fEc}(t)+I_{fc}V(t))}{N}-\delta_{f}*I_{cEf}(t)\\ \frac{dI_{fc}(t)}{dt}=\delta_{c}*I_{fEc}(t)+\delta_{f}*I_{cEf}(t)-\mu_{fc}*I_{fc}(t)-\omega_{fc}*I_{fc}(t)\\ \frac{dH_{f}(t)}{dt}=\mu_{f}*I_{f}(t)-\gamma_{f}*H_{f}(t)\\ \frac{dH_{c}(t)}{dt}=\mu_{c}*I_{c}(t)-\gamma_{c}*H_{c}(t)\\ \frac{dH_{fc}(t)}{dt}=\mu_{fc}*I_{fc}(t)-\gamma_{fc}*H_{fc}(t)\\ \frac{dR_{f}(t)}{dt}=\omega_{f}*I_{f}(t)+\gamma_{fc}*H_{fc}(t)-\sigma_{f}*R_{f}(t)\\ \frac{dR_{c}(t)}{dt}=\omega_{c}*I_{c}(t)+\gamma_{fc}*H_{fc}(t)-\sigma_{c}*R_{c}(t)\\ \frac{dR_{fc}(t)}{dt}=\omega_{c}*I_{c}(t)+\gamma_{fc}*H_{fc}(t)-\sigma_{fc}*R_{fc}(t)\\ N=S_{uv}+S_{vf}+S_{vc}+E_{f}+E_{c}+E_{fc}+I_{f}+I_{c}+I_{fEc}+I_{cEf}+I_{fc}+H_{f}+H_{c}+H_{fc}+R_{f}+R_{c}+R_{fc} \end{array}\right.$$

### 2.2 Model parameters

The model parameters mainly include the virus characters, as well as parameters related to the severity and recovery rates. Viral characters include the basic reproduction number and incubation period. Parameters related to severity consider the vaccination rates and are effective at preventing severe disease in both young and elderly populations. The epidemiological parameters are presented in **Table 1**.

**Table 1:** Summary of parameters.

Parameter Symbols	Parameter Description	Value		
		Whole population	Young people	Old people
v	Vaccination rate (%)	v_c: 89.7 (Liu et al., 2023) v_f: 2.47 (Zhao et al., 2022)	v_c: 89.7 (Liu et al., 2023) v_f: 0.65 (Wu, 2022)	v_c: 89.7 (Liu et al., 2023) v_f: 2.47 (Zhao et al., 2022)
e	Vaccine effectiveness (%) (against infection)	e_c: 80.5 (Chin et al., 2021) e_f: 50 (Rondy et al., 2017)	e_c: 85 (Liu et al., 2021) e_f: 80 (Hirve and W.H. Organization, 2015)	e_c: 79.49 (Li et al., 2022) e_f: 50 (Lyngse et al., 2021)
λ	Transmission rate (%)	λ_d: 38 (Lyngse et al., 2021) λ_b: 59.3 λ_x: 71.1 λ_ia: 35 (Fielding et al., 2015) λ_ib: 35 (Cowling et al., 2014)	λ_c: 38 (Lyngse et al., 2021) λ_f: 35 (Fielding et al., 2015)	
ε	Risk of infection from the other pathogen	ε_c: 1.58 ε_f: 1		
δ	Incubation period	δ_d: 4.4 (Zhang et al., 2021) δ_b: 2.6 (Tanaka et al., 2022) δ_x: 3 (Ogata and Tanaka, 2023) δ_ia: 3.4 (Pormohammad et al., 2021) δ_ib: 0.6 (Park and Ryu, 2018)	δ_c: 8.82 (Wu et al., 2022) δ_f: 1.4 (Park and Ryu, 2018)	δ_c: 4.4 (Zhang et al., 2021) δ_f: 3.4 (Pormohammad et al., 2021)
φ	Severity rate (%)	φ_c: 16.1 (Cheung et al., 2022) φ_f: 4.09 (Zheng et al., 2021) φ_fc: 13.89 (Zheng et al., 2021)	φ_c: 5 (Cheung et al., 2022) φ_f: 0.03 (Zheng et al., 2021) φ_fc: 13.89 (Zheng et al., 2021)	φ_c: 11.8 (Cheung et al., 2022) φ_f: 0.079 (Zheng et al., 2021) φ_fc: 13.89 (Zheng et al., 2021)
β	Vaccine protection rate against severe (%)	β_c: 97.4 (Zheng et al., 2022) β_f: 60.7 (Gras-Valentí et al., 2021) β_fc: 75 (Fernandes et al., 2022)	β_c: 75 (Fernandes et al., 2022) β_f: 60.7 (Gras-Valentí et al., 2021) β_fc: 75 (Fernandes et al., 2022)	β_c: 75 (Fernandes et al., 2022) β_f: 55 (Gras-Valentí et al., 2021) β_fc: 75 (Fernandes et al., 2022)
ω	Recovery period for mild cases	ω_d: 14 (Rees et al., 2020) ω_b: 5 (Menni et al., 2022) ω_x: 4 (Karyakarte et al., 2023) ω_ia: 6 (Moghadami, 2017) ω_ib: 7 (Moghadami, 2017)	ω_c: 14 (Faycal et al., 2022) ω_f: 5 (Ampomah and Lim, 2020) ω_fc: 30	ω_c: 18 (Faycal et al., 2022) ω_f: 14 (Ampomah and Lim, 2020) ω_fc: 30
γ	Recovery period for severe cases	γ_c: 20 (Tolossa et al., 2021) γ_f: 31.5 (Yazici Özkaya et al., 2022) γ_fc: 31.5	γ_c: 20 (Tolossa et al., 2021) γ_f: 15 (Yazici Özkaya et al., 2022) γ_fc: 65	γ_c: 40 (Tolossa et al., 2021) γ_f: 30 (Yazici Özkaya et al., 2022) γ_fc: 75
σ	Period of co-infection re-susceptibility due to immunity decrease	σ_c: 210 (Pilz et al., 2022) σ_f: 261 (Young et al., 2017) σ_fc: 261 (Altarawneh et al., 2023)		

The subscripts of (c) stand for COVID-19, (f)for Influenza, (fc) for Co-infection, Delta(d), BA.5(b), XBB(x), Influenza A(ia), Influenza B(ib).

### 2.3 Vaccination simulation

To capture the difference in VE against infection across age group groups, we divided the population (S) based on their vaccination status into protected by the COVID-19 vaccine (Svc), protected by the influenza vaccine (Svf), and unprotected by any vaccine (Suc). To estimate the number of populations protected by a vaccine, it is necessary first to identify the number of people vaccinated and then also to consider the effectiveness of the vaccine. Therefore, we introduce the following parameters: vaccination rate v and vaccine effectiveness against infection e.

For example, to estimate the number of populations protected by the influenza vaccine (Svf), we consider the proportion of the population vaccinated (vf) and the vaccine efficacy (ef), resulting in the following formula for Svf:


Svf=S×vf×ef

Similarly, the number of susceptible populations protected by the COVID-19 vaccine:


Svc=S×vc×ec

The model also incorporates the VE against severe cases. We consider the post-vaccination severe rate μ to be the remaining risk of developing severe illness excluding vaccine protection based on severe rate φ. The remaining risk of developing severe illness could be presented as “1 - β”.

Therefor μ is calculated as:


μ=φ*(1− β)

### 2.4 Sensitivity analysis

Our research employed PRCC to examine the relative impact of specific parameters on the simulation results in three distinct scenarios: influenza, COVID-19, and co-infection of influenza and COVID-19. The Partial Rank Correlation Coefficient (PRCC) serves as an essential sensitivity analysis tool, quantifying the influence of marginal changes in input parameters on the outputs of the Susceptible-Exposed-Infectious-Recovered (SEIR) model. A perfect linear relationship between the ranks yields a rank correlation coefficient of +1 (or -1 for a negative relationship) and no linear relationship between the ranks yields a rank correlation coefficient of 0. Partial rank correlation is the correlation between two variables after removing the effect of one or more additional variables.

## 3 Result

### 3.1 Co-infection of influenza and SARS-CoV-2

**Figure 2:**
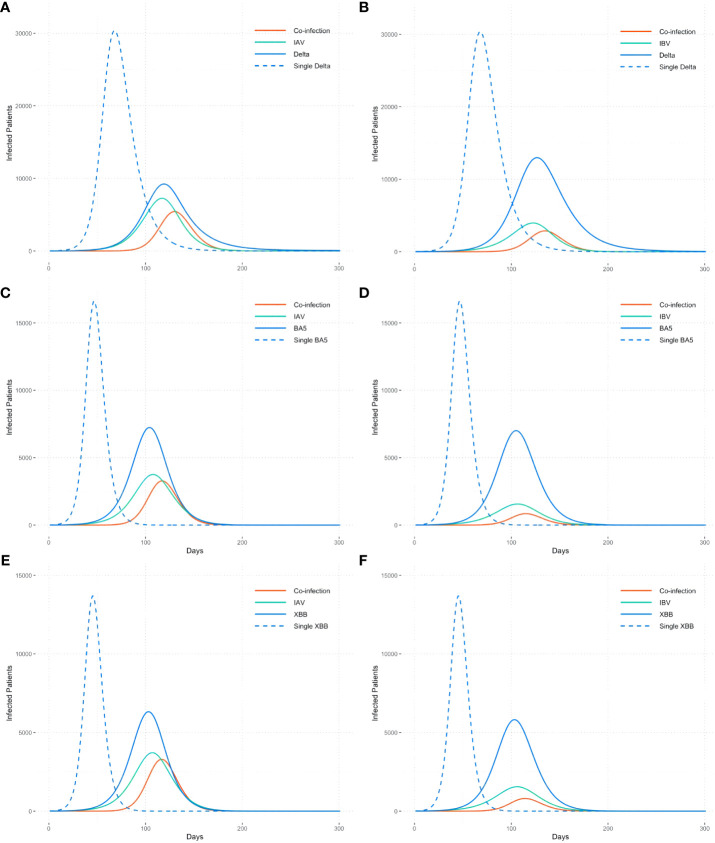
Co-epidemic of influenza types with different SARS-CoV-2 variants. **(A)**IAV+DELTA, **(B)**IBV+DELTA, **(C)**IAV+BA5, **(D)**IBV+BA5, **(E)**IAV+XBB, **(F)**IBV+XBB. The dotted line is the single epidemic curve of COVID-19, and the solid line refers to the number of co-infection and the trend of flu and COVID-19 when co-epidemic.

Due to the variety of influenza types and SARS-CoV-2 variants prevalent in different countries or regions globally, we have chosen the major influenza types (IAV, IBV) and SARS-CoV-2 variants (DELTA, BA.5, and XBB) for analysis. To elucidate the interactions between different types of viruses, we modeled six co-epidemic scenarios (**Figure 2**). Additionally, this study examines the impact of co-epidemic on SARS-CoV-2 spread, with the single epidemic in comparison, independently with identical initial infection and immune conditions.

Number of cases co-infected with IAV and SARS-CoV-2 is higher than that with IBV, approximately 2 times, demonstrating differences in interactions towards SARS-CoV-2 between different influenza virus genotype. For the same type of influenza virus, the number of co-infected cases increases as SARS-CoV-2 becomes more transmissible, like from the Delta strain to the Omicron strain (**Figures 2A, C, E**).

Compared to the single epidemic, the transmission curve of the SARS-CoV-2 exhibits a lower trend when co-epidemic with influenza. Meanwhile, the occurrence of co-infection leads to a delayed peak time of SARS-CoV-2, with an average delay of 57 days compared to the single epidemic.

### 3.2 Protective effect of vaccination against severe illness due to co-infection

To investigate the impact of vaccination on preventing patients with co-infection from severe, we vary the proportion of individuals vaccinated with the third dose of COVID-19 vaccine (vertical axis) with the proportion vaccinated against influenza (horizontal axis) and examine the impact on severe co-infection (Hfc).

**Figure 3:**
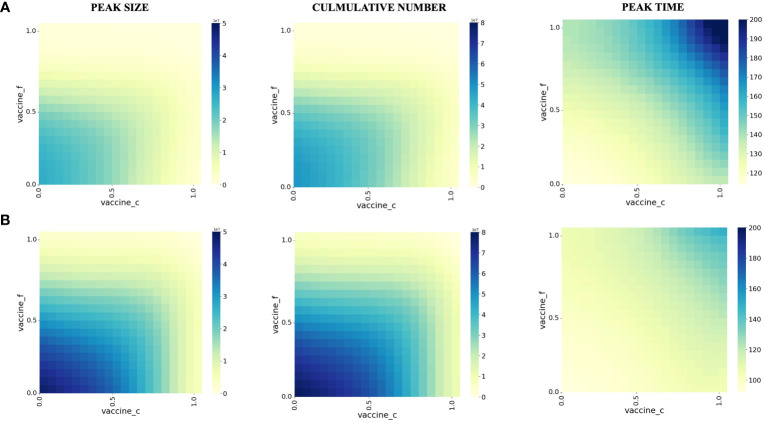
Effect of vaccination on peak size, peak time, and cumulative number of co-infection in different populations. **(A)** People younger than 60 years old years old **(B)** People older than 60 years old years old. The proportion of the susceptible population vaccinated against influenza during the initial pandemic period is shown on the vertical axis. The proportion vaccinated against COVID-19 is shown on the horizontal axis, and each square in the figure represents the modeled outcome of the model at the two current vaccination rates.

We plot some color-coded simulations to visualize the variation of the peak size, cumulative number, and peak time in **Figure 3**. Overall, as the proportion of individuals vaccinated with the COVID-19 vaccine and influenza vaccines increases, the peak number of co-infected severe illnesses and the number of severe illness cases decreases and the peak time is delayed.

We also took age-specific vaccine effectiveness into account, mainly for<60 (Panel A) and >60 years old (Panel B). For those<60 years old, the effectiveness of vaccine protection against severe illness is more pronounced in those >60 years of age. Based on our modeling results, for the< 60-year-old population, when the vaccination proportion of the population reaches at least 70% for COVID-19 and 55% for influenza, the cumulative number of co-infections and the number of severe illnesses is substantially reduced.

For those >60 years of age, the cumulative number of co-infections and the number of severe illnesses were substantially reduced when the vaccination proportion of the population reached at least 85% for COVID-19 and 70% for influenza.

### 3.3 Sensitivity analysis

**Figure 4:**
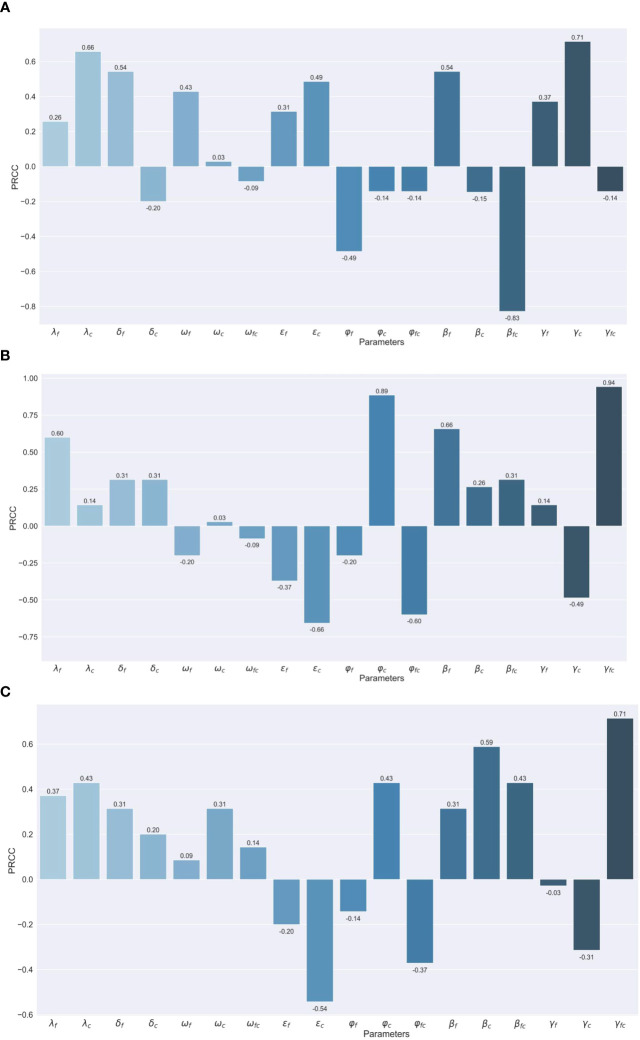
Assessing the Effects of Different Parameters on Co-infection Cases through PRCC Analysis **(A)** Maximum number of co-infection cases. **(B)** Peak Timing of co-infection cases. **(C)** Total number of co-infection cases.

We conduct the sensitivity analysis using the partial rank correlation coefficients (PRCC) method on different outcomes important to public health intervention, like peak number (**Figure 4A**), the peak time of co-infections (**Figure 4B**), and the total number of co-infections (**Figure 4C**), to identify the key parameters of the epidemic with the hope of determining public health measures that can be implemented to epidemic control and decreasing disease burden. We focus on parameters with the absolute value of PRCC greater than 0.06.

For the peak number of coinfections, the transmission rate of COVID-19 (indicated as ' $\lambda_{c}$ ') and recovery period for severe cases of COVID-19 (' $\gamma_{fc}$ ') have a positive impact, while the vaccine protection rate against severe co-infection (' $\beta_{fc}$ ') has a negative impact. The positive correlations suggest that with the escalation of the transmission rate of COVID-19 and the recovery period for severe cases of COVID-19, the peak size also experiences a notable increase. Conversely, the vaccine protection rate against severe co-infection is unique due to its considerable negative correlation. This implies that an improvement in the VE against co-infection from severe can potentially lead to a decline in such cases.

For the peak time for co-infection population, infection rates of influenza (' $\lambda_{f}$ ') severity rates for COVID-19 (' $\varphi_{f}$ ') the protection rate of the vaccine against severe influenza (' $\beta_{f}$ ') and the recovery period of severe co-infection cases (' $\gamma_{fc}$ ') ‘demonstrate a positive correlation with PRCC values of 0.60, 0.89, 0.66 and 0.94, respectively (**Figure 2B**). While susceptibility of influenza patients to COVID-19 (' $\varepsilon_{c}$ ') and severity rates of co-infection (' $\varphi_{fc}$ ') posed a negative correlation.

As to the total number of coinfections, severe co-infection cases (' $\gamma_{fc}$ ') are strongly positively correlated and susceptibility of influenza patients to COVID-19 (' $\varepsilon_{c}$ ') is in reverse.

## 4 Discussion

In this article, we developed the SEIHR model to simulate the co-prevalence of influenza viruses and SARS-CoV-2 to determine the impact on the health of the population and to facilitate the adoption of targeted strategies. At the same time, we elucidated the benefit of vaccination for co-infection populations based on age stratification.

WHO has declared an end to COVID-19 as a public health emergency, and some articles note that COVID-19 is now entering mini-waves rather than seasonal surges (Callaway, 2023). Reduced exposure due to PHSM during the COVID-19 pandemic resulted in insufficient preexisting immunity to influenza in the population, which could shift the immune landscape, build up a susceptible pool, and further alter the timing, trajectories, and severity of influenza in future seasonal epidemics (Ashraf et al., 2022). The above conditions make it much more likely that influenza and COVID-19 will be co-prevalent at the same time in the short term. It is important to raise awareness of co-infection with COVID-19 and influenza viruses and to assess the risk of co-infection manner promptly.

*In vivo*, experiments have demonstrated that infection with influenza causes a slight upregulation of ACE2 expression levels (2-3 folds), and that co-infection with influenza and COVID-19 strongly upregulates ACE2 expression levels (20 folds) (Swets et al., 2022), but the studies did not compare the differences between the different influenza virus types. Typically, influenza A and B viruses take turns to be the dominant types. The impact of different types of influenza and COVID-19 co-prevalent is still unknown. Our study suggests that influenza A, when co-prevalent with COVID-19, will cause a higher peak of co-infection than influenza B, which may be because influenza A viruses spread at a greater rate than influenza B (Lei et al., 2022; Yuan et al., 2013) and have a shorter incubation period (Zhong et al., 2011).

Existing studies have reported variable prevalence of co-infection. However, it is well recognized that co-infections can exacerbate damage in immunocompromised populations and significantly increase rates of severe illness and mortality. And it’s noted that simultaneous vaccination against H1N1 and SARS-CoV-2 is an effective prevention strategy for the coming winter (Bao et al., 2021). Our study found that simultaneous vaccination of populations against influenza and COVID-19 before the onset of the co-prevalent season did reduce the number and peak of cumulative severe illnesses, which effectively reduced the burden of disease and the impact on the healthcare system in co-infected populations. Moreover, the results of the age stratification of the model suggest that the increasing vaccination rate of people >60 years old is more effective in reducing and delaying the peak of severe illness, which suggests that areas with scarce vaccine resources may choose to prioritize vaccination of people >60 years old, At the same time, reducing the number of severe cases of co-infections, influenza vaccination seems more effective than COVID-19 vaccination, but the two vaccines are administered at similar rates, which encourages the development of bivalent vaccines.

Sensitivity analyses of the model showed that the number of peak co-infections was mainly positively correlated with the rate of transmission of the COVID-19 virus and the length of recovery from COVID-19 severe illness, and therefore the transmission and virulence of the COVID-19 epidemic strains should be continuously monitored. The protective effect of vaccines against severe disease from co-infections is a significant negative correlate, so timely assessment of the ability of virulent strains to escape from infection or vaccination to produce neutralization antibodies is also important.

In the construction of the model, we considered the infection order of the two viruses, the cross-protective effect of the vaccine, and the attenuation of the neutralization antibody, to restore as much as the influencing factors of co-infection in the population, providing a scientific basis for vaccine resource allocation as well. At the same time, we selected strains with potential pandemics. We presented scenarios of co-prevalence of different types of influenza viruses and SARS-CoV-2, which provides an assessment of the health risk of the population raised by coinfection. There are still some limitations in our study. Firstly, we only considered co-infection in the population under co-prevalence of COVID-19 and influenza. Therefore, we believe that there is no widespread co-infection in the population when the two viruses are not co-pandemic. Secondly, few data have been reported on the rate of protection against severe disease in co-infected patients by vaccination. Therefore, for parameters such as the vaccine protection rate against co-infection, we refer to the COVID-19 protection rate, as there are more real-world studies on this data. It is important to note that the model parameters utilize conventional seasonal parameters. However, due to previous large-scale outbreaks of COVID-19, the parameters for XBB may encompass the influence of population immunity levels.

## 5 Conclusion

To minimize the number of severe illnesses arising from co-infection of influenza and COVID-19, in conjunction vaccinations in the population are important, especially priority for the elderly.

## Data availability statement

The data that support these findings are available on reasonable request from the corresponding author. Data are not publicly available due to concerns regarding research participant privacy.

## Author contributions

JL: Conceptualization, Data curation, Investigation, Writing – original draft, Methodology. YW: Conceptualization, Methodology, Writing – original draft. ZL: Data curation, Writing – review & editing, Software. WH: Writing – review & editing, Investigation, Visualization. JS: Data curation, Writing – review & editing. QL: Data curation, Writing – review & editing. MZ: Data curation, Writing – review & editing. ZC: Data curation, Writing – review & editing. YG: Data curation, Writing – review & editing. WZ: Data curation, Writing – review & editing. TL: Data curation, Writing – review & editing. ZZ: Writing – review & editing, Supervision. CH: Funding acquisition, Supervision, Writing – review & editing. ZY: Writing – original draft, Funding acquisition, Supervision.
